# Gene coexpression clusters and putative regulatory elements underlying seed storage reserve accumulation in Arabidopsis

**DOI:** 10.1186/1471-2164-12-286

**Published:** 2011-06-02

**Authors:** Fred Y Peng, Randall J Weselake

**Affiliations:** 1 Agricultural Lipid Biotechnology Program, Department of Agricultural, Food and Nutritional Science, University of Alberta, Edmonton, Alberta, T6G 2P5, Canada

## Abstract

**Background:**

In Arabidopsis, a large number of genes involved in the accumulation of seed storage reserves during seed development have been characterized, but the relationship of gene expression and regulation underlying this physiological process remains poorly understood. A more holistic view of this molecular interplay will help in the further study of the regulatory mechanisms controlling seed storage compound accumulation.

**Results:**

We identified gene coexpression networks in the transcriptome of developing Arabidopsis (*Arabidopsis thaliana*) seeds from the globular to mature embryo stages by analyzing publicly accessible microarray datasets. Genes encoding the known enzymes in the fatty acid biosynthesis pathway were found in one coexpression subnetwork (or cluster), while genes encoding oleosins and seed storage proteins were identified in another subnetwork with a distinct expression profile. In the triacylglycerol assembly pathway, only the genes encoding diacylglycerol acyltransferase 1 (DGAT1) and a putative cytosolic "type 3" DGAT exhibited a similar expression pattern with genes encoding oleosins. We also detected a large number of putative *cis*-acting regulatory elements in the promoter regions of these genes, and promoter motifs for LEC1 (LEAFY COTYLEDON 1), DOF (DNA-binding-with-One-Finger), GATA, and MYB transcription factors (TF), as well as SORLIP5 (Sequences Over-Represented in Light-Induced Promoters 5), are overrepresented in the promoter regions of fatty acid biosynthetic genes. The conserved CCAAT motifs for B3-domain TFs and binding sites for bZIP (basic-leucine zipper) TFs are enriched in the promoters of genes encoding oleosins and seed storage proteins.

**Conclusions:**

Genes involved in the accumulation of seed storage reserves are expressed in distinct patterns and regulated by different TFs. The gene coexpression clusters and putative regulatory elements presented here provide a useful resource for further experimental characterization of protein interactions and regulatory networks in this process.

## Background

Seed storage reserves accumulated during embryogenesis in higher plants are crucial for plant propagation, providing carbon and energy during germination prior to seedling establishment. In mature Arabidopsis seeds, storage lipids and proteins are the major storage compounds, each accounting for 30% - 45% of the seed dry weight [1]. The past decade has witnessed a substantial progress in identification and characterization of genes involved in the *de novo *fatty acid (FA) biosynthesis and triacylglycerol (TAG) assembly pathways [[1,4] and references therein]. This advancement is particularly evident in the model plant Arabidopsis, largely owing to the sequencing and release of its relatively compact genome in the year 2000 [5]. Moreover, characterization of transcription factors (TFs) has led to the identification of several master regulator genes that play critical regulatory roles in this biological process, including *ABI3 *(*ABSCISIC ACID INSENSITIVE 3*), *LEC1 *(*LEAFY COTYLEDON 1*), *LEC2 *and *FUS3 *(*FUSCA 3*) [6-17]. These TFs interact with each other and form complex regulatory networks [18-23], regulating multiple aspects of seed development including storage reserve accumulation through interaction with cognate *cis*-acting DNA elements in the promoter regions of target genes. ABI3, FUS3 and LEC2 contain a plant-specific 'B3' DNA-binding domain which targets RY-repeat regulatory elements, whereas LEC1 and L1L (LEC1-LIKE) contain a NF-YB domain binding to the CCAAT boxes in the promoter region [24,25]. Additional TFs such as WRINKLED 1 (WRI1), a member of plant-specific APETALA 2 (AP2) - ethylene response element binding factor (EREB) family, is also known to control transcription of many FA biosynthetic genes [26], and recent studies show it acts via binding to the AW-box motif present in the promoter region of 19 FA biosynthetic genes [27]. Moreover, ABI4 (an AP2 family protein) and various basic-leucine zipper (bZIP) TFs including ABI5 or EEL (ENHANCED EM [EMBRYO MORPHORGENESIS] LEVEL) are known regulators of the expression of *SEED STORAGE PROTEIN *(*SSP*) genes, which act in the same signalling network but downstream of ABI3 [28,29]. Distinct regulatory mechanisms are present in controlling the accumulation processes of oils and proteins, perhaps with cross-talk to coordinate the synthesis of seed storage compounds. This coordination could help to explain the well-documented negative correlation (correlation coefficient ranging from -0.60 to -0.90) between oil and protein content in seeds of many oleaginous species [[3] and references therein]. Moreover, several TFs, such as LEC1, ABI3 and FUS3, have been demonstrated to regulate many genes in the synthesis of both oils and storage proteins in developing seeds [30-32].

In contrast to the great advancement in characterizing individual genes involved in the accumulation of seed storage reserves, the relationship of their expression and regulation is not well understood. A more holistic view of this biological process at the systems level would prove beneficial in developing strategies to further enhance seed yield and oil content, as well as in the modification of oil composition. To gain insights into global transcriptional dynamics in key cellular processes, microarray is an effective method for analyzing the transcript abundance of a large number of genes simultaneously. Datasets obtained from profiling experiments can be further used to infer gene regulatory networks. In Arabidopsis, two cDNA microarrays were designed several years ago based on the expressed sequence tag (EST) sequences available at the time. One array was used for tissue-specific expression profiling to identify genes that are preferentially expressed in developing seeds compared with vegetative leaves and roots [33], and the other was used to study the temporal pattern of gene expression during the critical period of seed filling [34]. These transcriptional profiling studies in Arabidopsis seeds have greatly increased our understanding of overall alterations of gene expression during seed development and storage reserve accumulation. These two early cDNA-based microarrays, however, surveyed <3500 unique Arabidopsis genes.

More recently, Schmid et al. [35] created a global gene expression atlas AtGenExpress (Expression Atlas of Arabidopsis development) representing the Arabidopsis life cycle using the Arabidopsis ATH1 genome array (Affymetrix, Santa Clara, CA), which can measure nearly 24,000 genes in a single assay. In AtGenExpress, 237 chips were hybridized for 79 different samples collected from various organs, growth stages and under various environmental conditions, including 24 arrays for eight stages of maturing seeds. Since its release, this exceptionally large transcriptome dataset has been a goldmine for plant biologists to identify candidate genes for molecular characterization. A number of studies have further "mined" this dataset within different contexts of plant biology. Wang et al. [36] extracted the expression data for several TFs experimentally determined to regulate seed development and genes that code for enzymes in the FA biosynthesis pathway. Volodarsky et al. [37] utilized the dataset to analyze hormone-related transcriptional activities in Arabidopsis. Vandepoele et al. [38] constructed coexpression networks and predicted *cis*-regulatory elements for the cell cycle-related TF OBP1. Recently, the identification of gene coexpression networks has emerged as a popular method for predicting gene functions and interactions [38-41], and web-based tools such as Genevestigator [42] and CressExpress [43] have been developed to facilitate such analyses at a small scale for plant biologists. Transcriptional coordination, or coexpression, of genes may be an indication of functional relatedness, based on the "guilt-by-association" principle [44]. In a coexpression network, a vertex or node represents a gene whereas an edge is a connection inferred from the correlation coefficient calculated from the gene expression data. Although the relationship between coexpression networks and true biological networks is often not clear, it has been shown that gene groups identified from modular (cluster) analysis in coexpression networks often exhibit an enrichment of certain Gene Ontology (GO) categories [45], suggesting the functional association of genes connected in a coexpression network. Hence, a coexpression edge can be considered a putative interaction between two genes. Genes in a coexpression network, particularly those expressed in a specific tissue or sharing a semantic similarity in the GO 'Biological Process' aspect, might be co-regulated through common TF binding sites in their upstream regions, leading to many attempts to identify overrepresented *cis*-motifs in coexpressed genes [46-50].

In the current study, we took advantage of this public transcriptome dataset in Arabidopsis [35], analyzed the raw data thoroughly in the context of seed storage reserve accumulation during seed development, and constructed coexpression networks for seed-expressed genes. We focused on genes involved in FA biosynthesis and the accumulation of storage lipids and proteins in developing seeds. This comprehensive analysis has resulted in the identification of a large number of genes that are possibly coexpressed and function cooperatively during seed maturation. Furthermore, we predicted a large number of *cis*-regulatory elements for key seed-expressed genes. This information could be useful in designing experiments to probe regulatory mechanisms underlying seed storage reserve accumulation.

## Results and Discussion

### Association of seed transcriptome with embryo morphology in developing Arabidopsis seeds

Using the raw intensity data generated by AtGenExpress for a global gene expression atlas throughout the Arabidopsis life cycle [35], we performed a detailed analysis of gene expression pertaining to seed storage reserve accumulation during the eight stages of seed development, ranging from globular embryo to mature embryo stages (Table 1). Of the nearly 24,000 genes represented on the Affymetrix GeneChip ATH1 genome array, we estimated that approximately 12,353 genes (or ~54%) were expressed in at least one of the eight development stages. Our analysis took into account the fact that certain genes might be transiently expressed at only one stage during seed development. The relatively high log2 intensity value of 6.0 was chosen as the threshold to focus on the genes with at least a modest level of expression. The global transcriptional activity in the developing Arabidopsis seed is higher than in the leaf, lower than in the flower, and comparable to that in the apex, root or stem (data not shown).

**Table 1:** Arabidopsis developing seed samples used for AtGenExpress microarray experiments.

Stage	Sample name	Tissue source	Stage description	Description
S3	Col-0_sil3	Seeds stage 3 with siliques	C globular stage	Mid globular to early heart
S4	Col-0_sil4	Seeds stage 4 with siliques	D bilateral stage	Early heart to late heart
S5	Col-0_sil5	Seeds stage 5 with siliques	D bilateral stage	Late heart to mid torpedo
S6	Col-0_seed6	Seeds stage 6 without siliques	E expanded cotyledon stage	Mid torpedo to late torpedo
S7	Col-0_seed7	Seeds stage 7 without siliques	E expanded cotyledon stage	Late torpedo to early walking-stick
S8	Col-0_seed8	Seeds stage 8 without siliques	E expanded cotyledon stage	Walking-stick to early curled cotyledons
S9	Col-0_seed9	Seeds stage 9 without siliques	F mature embryo stage	Curled cotyledons to early green cotyledons
S10	Col-0_seed10	Seeds stage 10 without siliques	F mature embryo stage	Green cotyledons

The descriptions of samples used for microarray experiments in AtGenExpress [35] were obtained from the TAIR where the raw data files were retrieved [71]. The development stages for these seed samples range from four to 12 days after pollination, encompassing the accumulation phase of both oils and storage proteins [1].

**Figure 1:**
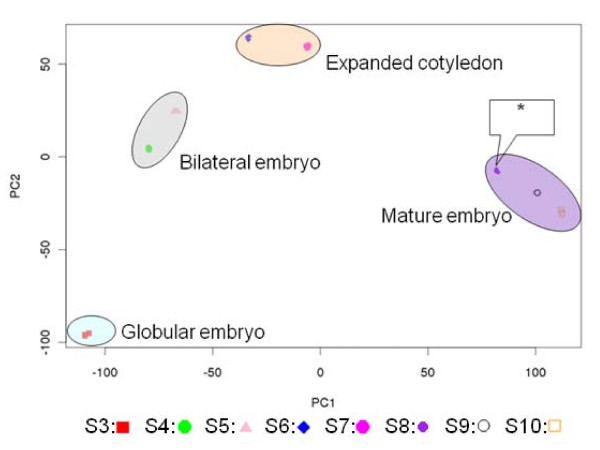
**The transcriptome dynamics during Arabidopsis seed development**. The normalized, log_2_-transformed expression data for the 24 samples were subjected to principal component analysis (PCA) using the R prcomp function [83]. PC1 and PC2 are the first two principal components in the dataset. Different symbols and colours shown at the bottom of the figure were used for different seed developmental stages to show the relationship between molecular and morphological phenotypes. As in Table 1, the different samples are as follows: S3: C globular stage; S4: D bilateral stage; S5: D bilateral stage; S6: E expanded cotyledon stage; S7: E expanded cotyledon stage; S8: E expanded cotyledon stage; S9: F mature embryo stage; S10: F mature embryo stage.

To examine the overall transcriptome changes across the eight seed development stages, we performed a principal component analysis (PCA) in the 'Sample' space, and the results indicated that the global transcriptional program changes constantly during seed maturation (Figure 1). In PCA, the first principal component (i.e., development stage) was estimated to explain ~83% of variance in the seed transcriptome, indicating that embryogenesis is the predominant cause for the substantial variation observed in the transcript population. The differences in the global gene expression patterns among the eight developing stages were cross validated by a global association test [51], showing that the seed transcriptome varied across the eight developmental stages in a statistically significant manner (*P *< 0.0001). The presence of siliques in the young seeds (S3 to S5; Table 1) may have had an effect on global transcript profiles in the seeds of earlier development stages, but its minor effect cannot be dissected from that of seed development under the experimental design in [35]. Additionally, Figure 1 shows that each stage has a distinct transcriptome signature that generally corresponds to its seed development stage defined by the embryo's morphology. For instance, as shown in Figure 1, the globular embryo stage (with three replicates) grouped tightly, the two samples from the bilateral stage clustered together but separately from other stages, and in general, samples from the expanded cotyledon stage and the mature embryo stage also clustered corresponding to their morphological stages, respectively. The transcriptome signature for one expanded cotyledon stage (with an asterisk in Figure 1), however, was closer to the two samples of the mature cotyledon stage, rather than the expanded cotyledon stage defined by embryo morphology. This result suggests that staging of seed development based on the embryo's morphological shape alone may not necessarily reflect the transcriptome state in the seed, which is attributable to the fact that molecular events, such as gene expression, occur prior to morphological changes. Consistent with the highly dynamic landscape in global gene expression, our analysis on individual genes using the method in [52] indicated that nearly all the genes expressed in developing Arabidopsis seeds are differentially transcribed under a stringent false discovery rate (FDR) threshold of 0.01 (data not shown). This lack of stably expressed genes with adequate transcript abundance brings into focus the challenge of determining reference genes that can be used for normalization in quantifying mRNAs in developing seeds [53]. In summary, this analysis demonstrates that the transcriptional program is subject to constant alterations during seed development as many other studies have shown, suggesting its tight regulation at the transcriptional level.

### Construction of gene coexpression networks in the Arabidopsis seed transcriptome

**Figure 2:**
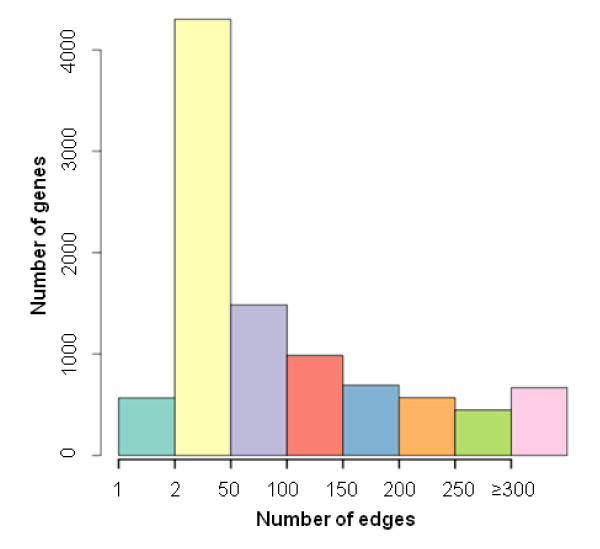
**Summary of the gene coexpression network in developing Arabidopsis seeds**. Distribution of the number of genes in different bins of edge numbers in the coexpression network of seed-expressed genes. The edge numbers were divided into different ranges and the frequency of nodes in each bin was found to summarize the coexpression network. The bin categories are as follows: 1; 2 - 49; 50 - 99; 100 - 149; 150 - 199; 200 - 249; 250 - 299; and ≥300.

To infer the gene coexpression network in the transcriptome of developing Arabidopsis seeds, we focused on the 12,353 genes with moderate or high expression levels. The Pearson-based correlation coefficient was used as a measure of expression coherence, and we applied a correlation threshold of 0.90 and retained over 1.7 million correlated gene pairs representing 11,698 distinct genes. The resulting coexpression networks encompassed approximately 95% of seed- expressed genes, indicating that the majority of expressed genes in Arabidopsis seeds act in a concerted manner. We chose such a stringent correlation threshold considering the relatively small sample size in the analysis so that gene pairs in the coexpression network are statistically significant (*P *= 0.0005 using Fisher's Z transformation), meaning the probability of randomly obtaining a correlation coefficient of ≥ 0.90 in this seed transcriptome dataset is small. The frequency distribution of the number of connections is shown in Figure 2. Nayak et al. [40] used the absolute correlation (|r|) to construct a gene coexpression network in human immortalized B cells, but we believe that positive and negative correlations in gene expression may indicate different biological interactions (synergistic or antagonistic), and therefore we only included gene pairs with positive correlation coefficients above the threshold for the coexpression analysis. Nevertheless, gene pairs consistently expressed in a negatively correlated manner can also be of great interest to biologists.

**Figure 3:**
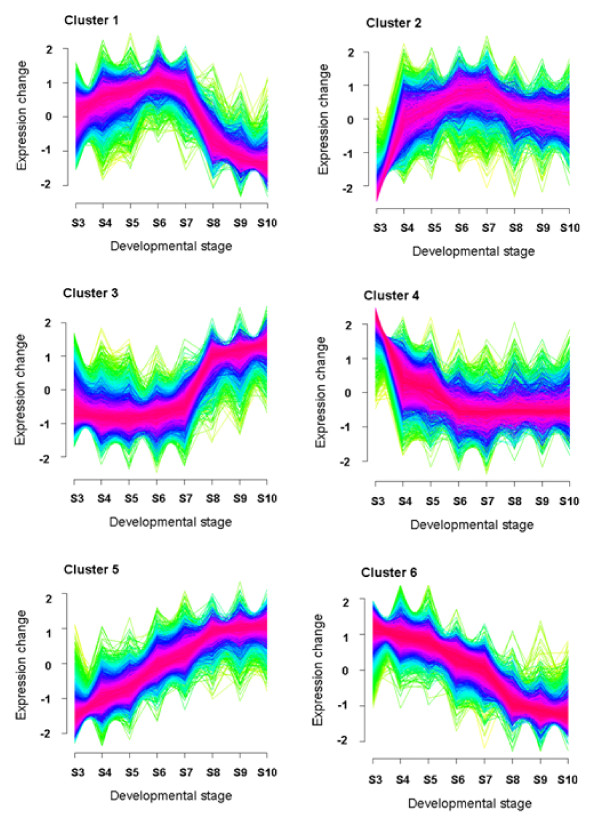
**Fuzzy clustering of the expression data along seed development series**. The six clusters showing the expression patterns during Arabidopsis seed development. The gene expression values were standardized to have a mean value of zero and a standard deviation of one for each gene profile. The transformed expressions were then clustered using the fuzzy c-means (FCM) clustering algorithm implemented in the Bioconductor Mfuzz package [89]. Based on preliminary analysis, we found six clusters can well represent different expression patterns inherent in the dataset, and another FCM parameter m = 1.75. A membership value in the range of 0-1 was assigned in clustering and the cluster cores consisting of genes with membership value > = 0.90 were coloured pink.

We also used a complementary clustering approach to identify gene clusters with similar expression profiles during seed maturation (Figure 3). We found six clusters could sufficiently represent the distinct patterns inherent in this seed transcriptome dataset, with some clusters being the "mirror images" of others. The first two clusters included the majority of genes related to the accumulation of seed storage reserves, which will be described in more detail below. It is important to point out that the method for identifying coexpression networks is computationally similar to various clustering approaches, using correlation coefficient (r) as the similarity measure, or alternately 1 - |r| as the distance measure. An important difference exists, however, in the parameters used in the two processes: the number of clusters is often specified in clustering although certain assessment can be performed beforehand, whereas the correlation threshold is chosen in the coexpression network analysis. We believe our approach of coexpression network identification, coupled with clustering, is advantageous for identification of genes in the same coexpression cluster with visible expression patterns during seed maturation, enabling easier biological interpretation and various complementary analyses.

Several parameters can be used to describe a biological network, including the clustering coefficient and scale-free topoplogy criterion. The scale-free topology criterion ranges from zero to one for typical biological networks under investigation [54-56]. The clustering coefficient and scale-free topology criterion were 0.73 and 0.68, respectively, in this Arabidopsis seed coexpression network (Table 2), indicating topological similarity to other biological networks. As shown in Figure 2, the network is comprised of many genes with few links (e.g., most genes have two to 100 putative coexpression partners) but relatively few genes with many connections, which is consistent with the power-law distribution widely present in biological networks. In the coexpression network, each gene has a median of 71 edges. It is notable that a relatively large number of genes have ≥300 edges (Figure 2), which is at least partly due to this larger range containing all remaining numbers of connections. We observed the edge numbers for genes in different Gene Ontology (GO) 'Biological Process' categories and did not find any association between the number of coexpression partners and obvious functional significance (data not shown); TF gene *LEC1 *and a ribosomal protein S18 gene (*RPS18*), for instance, were found to connect with 38 and 178 coexpression partners, respectively. This indicates that, while the number of edges for a node may suggest the functional significance of the gene, the centrality (or location) of a node in the network can be more important. This aspect has been well described in social network analysis [57].

**Table 2:** Network characteristics in the Arabidopsis seed coexpression network.

Total number of genes in the network	11,698
Mean number of connections per gene	160

Median number of connections per gene	71

Maximum connections	367

Clustering coefficient ^a^	0.73

Scale-free topology criterion ^b^	0.68

Gamma ^c^	1.34

Network properties were analysed according to the methods in [40]. ^a ^The clustering coefficient measures the "small-world" property in the network, which is the likelihood that two genes connected to a common gene are also connected to each other [54]. ^b ^The scale-free topology criterion is used to measure the topological similarity of a network to other biological networks. Its value ranges from 0 to 1, with 1 representing networks that are most like other biological networks [55]. ^c ^Gamma is the measurement of power-law distribution in a network [56], which consists of many genes with relatively few connections and a few genes (hubs) with many connections; a gamma < 3 indicates that a network exhibits such a distribution [40].

### Genes encoding fatty acid biosynthetic genes and seed storage reserve associated proteins are located in different subnetworks

**Figure 4:**
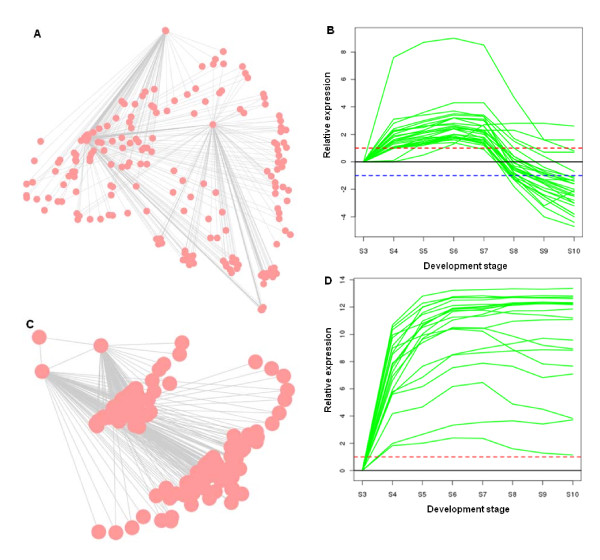
**Subnetwork and temporal expression profiles for genes involved in seed storage reserve accumulation in developing Arabidopsis seeds**. A is the subnetwork for genes including those in fatty acid (FA) biosynthesis, and B depicts the expression profiles of FA biosynthetic genes identified in the analysis. C is another subnetork including genes encoding oleosins and seed storage proteins (SSP), and D depicts the expression profiles of genes encoding oleosin and SSP. In B and D, the expression values, AGI identifiers of the genes depicted are listed in Additional File 3, and the log_2 _expression values were standardised by subtracting the value at the first S3 stage for each gene. Dashed red, blue lines indicate 2-fold up- or down-regulation, respectively.

**Figure 5:**
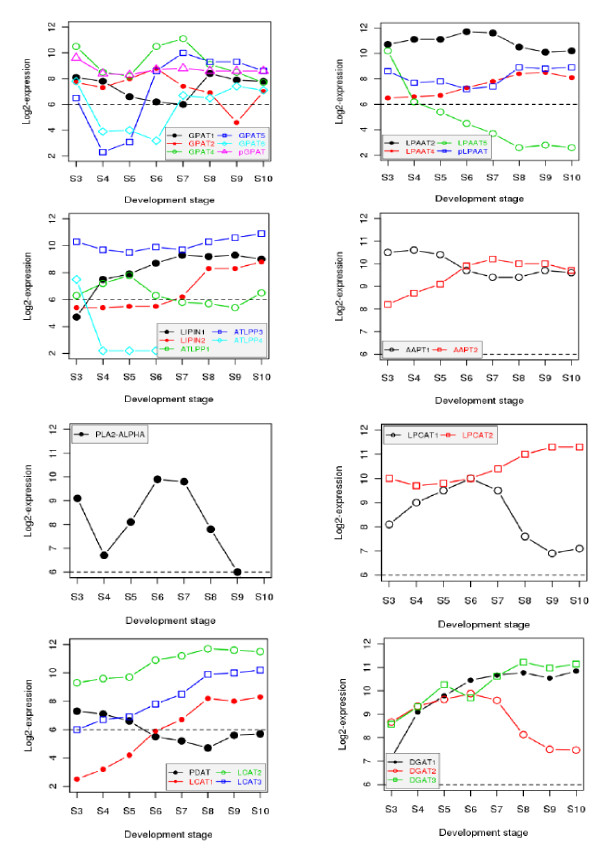
**Expression profiles of genes including homologues in the triacylglycerol assembly pathway**. The dash line at 6.0 is often used as the cutoff for present (expressed; above the line) or absent (unexpressed; below the line). All expression data were transformed to the log_2 _scale for plotting the profiles. Genes and homologs in the triacylglycerol (TAG) assembly pathway were identified based on an early survey of Arabidopsis genes involved in acyl lipid metabolism [59], and their AGI identifiers listed in Additional File 3. Refer to [64] for their roles in TAG assembly. The abbreviations of these genes and their encoded enzymes (EC numbers) are as follows: *GPAT*: *sn*-glycerol-3-phospahte acyltransferase (EC 2.3.1.15); *LPAAT*: lysophosphatidic acid acyltransferase (EC 2.3.1.15); *PAP*: PA phosphatase (EC 3.1.3.4), including *LIPIN *(*PAP1*) and *LPP *(*PAP2*); *AAPT*: Aminoalcoholphosphotransferases (EC 2.7.8.1 and EC 2.7.8.2); *CPT*: cytidine diphosphate (CDP)-choline: 1, 2-diacylglycerol cholinephosphotransferase (EC 2.7.8.2); LPCAT: lysophosphatidylcholine acyltransferase (EC 2.3.1.23); *PLA*_*2*_: Phospholipase A_2 _(EC 3.1.1.4); *PDAT*: phospholipid:diacylglycerol acyltransferase (EC 2.3.1.158); *LCAT*: lechitin:cholesterol acyltransferase (EC 2.3.1.43), these three shown here are *PDAT *homologs; *DGAT*: Diacylglycerol acyltransferase (E.C. 2.3.1.20).

While the entire coexpression network is useful for network topology analysis, isolation of a subnetwork (or cluster) makes it more accessible to biologists [40,58]. More importantly, a subnetwork in the large coexpression network is often more biologically relevant in a pathway context. Hence, we extracted subnetworks from this gene coexpression network for genes relevant to the accumulation of seed storage reserves (Figure 4). Of the 48 genes known to encode enzymes involved in FA biosynthesis [17,59], we identified 44 (or ~92%) genes represented on the ATH1 array, and all of them were found in one subnetwork (Figure 4A). This subnetwork cluster consists of 1854 genes (Additional File 1), which is in general agreement with an interactive correlation network generated genome-wide in Arabidopsis using a heuristic clustering algorithm [41]. Such a gene list can be used to identify interactors of genes in FA synthesis in developing seeds. Consistent with the coexpression subnetwork analysis, the majority of genes involved in FA biosynthesis were associated with Cluster 1 (Figure 3). Their expression levels increased steadily from the globular embryo stage, generally reached the peak at the expanded cotyledon stage, and dramatically declined subsequently throughout late seed maturation (Figure 4B). Such a unified expression pattern for most FA biosynthetic genes supports earlier studies showing that FA supply can be a limiting factor for triacylglycerol (TAG) accumulation in developing embryos of *Brassica napus *[60], olive (*Olea europaea *L.) and oil palm (*Elaeis guineensis *Jacq.)[61], as well as *cuphea lanceolata *and other oil species [62]. Recent studies of metabolic flux in developing embryos of *B. napus*, however, indicated that TAG assembly was more limiting than FA biosynthesis in regulating the flow of carbon into TAG [63]. The majority of genes encoding oilbody oleosins and SSPs were found in another subnetwork with a distinct expression pattern (Figure 4C). The subnetwork encompassing genes encoding oleosins and SSPs is comprised of 1392 genes (Additional File 2). Genes encoding oleosins and SSPs were in Cluster 2 (Figure 3), and their expression profiles were strikingly similar. These genes were virtually unexpressed at the globular stage, increased rapidly (>1000-fold in many cases) from the globular stage to the bilaternal stage, and remained at the elevated expression level throughout the remaining stages of seed maturation (Figure 4D). Transcripts for *OLEOSIN *and *SSP *genes are most abundant in the seed transcriptome late during seed development. In contrast, most genes in the TAG assembly pathway were found in different subnetworks, exhibiting various expression profiles during seed development (Figure 5). *DIACYLGLYCEROL ACYLTRANSFERASE 1 (DGAT1*), *FATTY ACID DESATURASE 2 (FAD2*), *FATTY ACID ELONGASE 1 *(*FAE1*) and *STEAROYL DESATURASE *(*SAD*) genes were identified in this subnetwork, albeit expressed at substantially lower levels compared to genes encoding oleosins and SSPs (Additional File 3). DGAT catalyzes the acyl-CoA-dependent acylation of *sn*-1,2-diacylglycerol to produce TAG and CoA [64]. FAD2 catalyzes the introduction of a second double bond into acyl groups in phospholipid whereas SAD catalyzes the formation of monounsaturated FA in the plastid [65]. FAE1 catalyzes the elongation oleoyl-CoA in the endoplasmic reticulum [65]. Our analysis determined that AT1G48300, which was named *DGAT3*, is the putative gene encoding a cytosolic DGAT in Arabidopsis. The amino acid sequence of AT1G48300 has a significantly high degree of similarity (expect value < 1 × 10^-21^) to the soluble DGAT in peanut (*Arachis hypogaea*), where the cytosolic *DGAT *gene in plants was first discovered [66]. Notably, *DGAT3 *exhibited a similar expression pattern with *DGAT1*, but expressed higher during late seed maturation. In earlier studies, quantification of DGAT activity during seed maturation in *B. napus *indicated that enzyme activity was maximal during the rapid phase of oil accumulation with a substantial decrease in activity occurring as oil levels reached a plateau [67,68]. Assuming DGAT activity shows a similar profile during seed development in Arabidopsis, this suggests that DGAT may be down-regulated post-transcriptionally and/or post-translationally during the latter stages of seed development.

In summary, our new results suggest that genes acting in a biological process (FA biosynthesis) can be indicated by their presence in the same coexpression network cluster, but genes involved in the same pathway (TAG assembly) may not necessarily exhibit expression coherence. As a result, computational approaches using coexpression network to predict gene function, such as in [40], will undoubtedly have limitations.

### Putative regulatory elements underlying seed storage reserve accumulation

**Figure 6:**
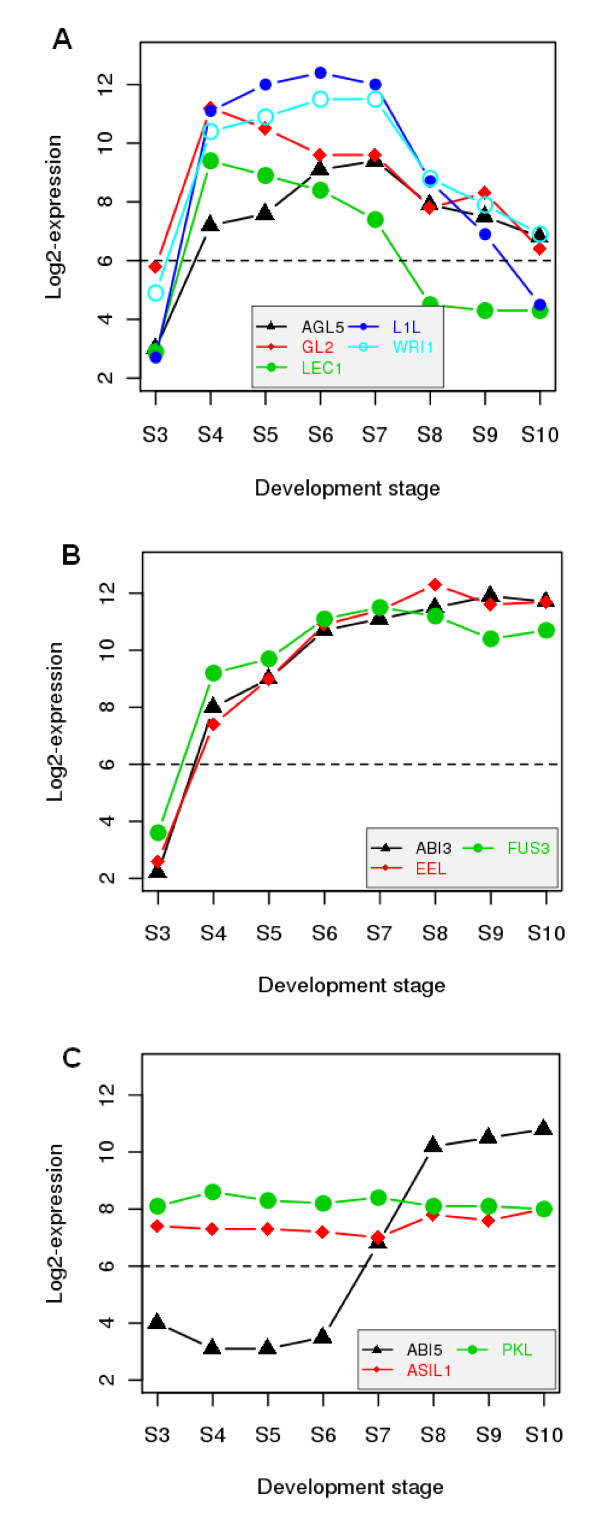
**Expression profiles of several well-characterized transcription factor genes**. The dashed line at 6.0 on the y-axis is often used as the cutoff for present (expressed; above the line) or absent (unexpressed; below the line). All expression data were transformed to the log_2 _scale for plotting the profiles. Refer to Additional File 3 for their AGI identifiers and full name of each transcription factor gene. The gene abbreviations are as follows: *AGL5: AGAMOUS-LIKE 5*; *GL2: GLABRA 2*; *LEC1: LEAFY COTELYDON 1*; *L1L: LEAFY COTELYDON 1 LIKE*; *WRI1: WRINKLED 1*; *ABI3: ABSCISIC ACID-INSENSITIVE 3*; *EEL: ENHANCED EM (EMBRYO MORPHOGENESIS) LEVEL*; *FUS3: FUSCA 3*; *ABI5: ABSCISIC ACID-INSENSITIVE 5*; *PKL: PICKLE*; *ASIL1: ARABIDOPSIS 6B-INTERACTING PROTEIN 1-LIKE 1*.

To gain insight into possible relationships in gene coexpression and regulation, we first identified the expression patterns for several TFs known to regulate the accumulation of seed storage reserves (Figure 6). *AGL15 (AGAMOUS-LIKE 15)*, *GL2 (GLABRA2)*, *LEC1*, *L1L*, and *WRI1 *exhibited similar expression patterns with most genes encoding proteins involved in FA biosynthesis (Figure 6A) whereas *ABI3, EEL*, and *FUS3 *all have similar expression profiles with genes encoding oleosins and SSPs (Figure 6B). Two repressors of seed maturation genes, *ASIL1 *(*ARABIDOPSIS 6B-INTERACTING PROTEIN 1-LIKE 1*) [69] and *PICKLE *(*PKL*) [70], were modestly expressed and exhibited a stable expression pattern throughout seed maturation (Figure 6C). Surprisingly, *LEC2*, a TF gene known to regulate oil accumulation in leaves and somatic embryogenesis [10,14,16], was barely detectable in these developing seeds. Although this result requires verification with other molecular methods, it was previously reported that *LEC2 *might be only expressed during early embryo morphogenesis [15]. Additionally, based on phenotype descriptions of *LEC1*, *LEC2 *mutants in the Arabidopsis Information Resource (TAIR) [71], the accumulation of storage compounds in the mature *lec2 *mutant seeds is only slightly defective when compared to *lec1 *mutant seeds. Therefore, the role of *LEC2 *as a regulator in the synthesis of seed storage reserves during late stages of zygotic embryo development might not be as important as currently thought. Likewise, *ABI4 *was also essentially unexpressed in these seed samples. The expression similarity between genes encoding TFs and their target genes is suggestive of regulatory relationships. Both *LEC1 *and *WRI1 *were clustered with most FA biosynthetic genes, while *ABI5 *was clustered with the majority of *LATE-EMBRYOGENESIS ABUNDANT *(*LEA*) genes (Figure 3 Cluster 3). *LEC1 *and *WRI1 *are known to regulate many FA biosynthetic genes [25-27], and *ABI5 *regulates a subset of *LEAs *[72].

To computationally identify *cis*-acting regulatory elements, the upstream promoter sequences for the genes involved in storage reserve biosynthesis were extracted from the RSAT server [73]. We included some 5'-UTR sequences as certain TF binding sites can be located within this region of a gene [27,74]. On average, the G-C content in the promoter sequences of the gene set was found to be <35%, which is consistent with the compositional bias of nucleotides towards A-T enrichment observed in plant promoter regions [74,75]. Two software tools, TFBS [76] and fdrMotif [77], were used to search for putative TF-binding sites on both strands. Both tools depend on TF- binding profiles (Position Weight Matrix, or PWM) derived from experimentally determined binding sites for the prediction, we thus compiled 118 PWMs from the literature [27,74] and the JASPAR database [78] (Additional File 4). In the JASPAR database, we only considered the binding profiles for plant-specific TFs because of their potential critical roles in regulating the accumulation of storage reserves during seed development, a unique physiological process in higher plants.

We predicted a total of 1770 binding motifs in the promoter regions of genes involved in FA biosyntheis, TAG assembly, and genes encoding oleosins and seed storage proteins (Additional File 5). Each TF can have more than one putative binding site in each gene. As our approach of using two predictive tools already filtered out a large number of potentially false predictions, the remaining number of putative motifs was relatively small, making it difficult to perform statistical analysis of motif enrichment. Therefore, we used a simple approach to determine overrepresentation of a TF binding motif in the gene set, and defined the number of the motifs for a particular TF as overrepresented if it is greater than the sum of the average plus the standard deviation of all predicted motifs in a gene set. Sequence logos are used to show the degree of conservation, indicated by the height of each nucleotide, at each position (Table 3). For the Aw-box motif interacting with WRI1, which possesses a sequence pattern of [CnTnG](n)_7_[CG] (where n is any nucleotide), we predicted binding sites in 26 of 44 FA genes identified, seven more than reported recently in [27]. The highly conserved CCAAT motifs for LEC1 (and L1L) binding are significantly enriched in promoters of all FA biosynthetic genes identified. Motifs that interact with TF genes known to regulate light-induced genes, such as Zinc-finger proteins DOF1 (MNB1A) and DOF2 [79], as well as GATA TFs and SORLIP 5 (Sequences Over-Represented in Light-Induced Promoter 5) [80], are overrepresented in the promoters of FA biosynthetic genes. *DOF1 *(AT1G51700), however, was expressed only at the early globular embryo stage. *DOF2 *(AT4G38000) exhibited a similar expression profile during seed development as for FA biosynthetic genes (data not shown). *ARR *(Arabidopsis Response Regulator) genes encode ARR7 and ARR15, which have been shown to regulate the interaction of cytokinin and auxin in root stem-cell specification during early embryogenesis [81]. We found no binding matrices for these two regulators, but the binding matrix for ARR10 is present in our compiled matrix set and ARR10 motifs are overrepresented in the promoter regions of FA biosynthetic genes. We also found no binding matrices for AGL 5 or GL2; binding profiles for AGL 3 and AGL 15 were present in our analysis but no enriched motifs were identified in the promoter sequences of these FA biosynthetic genes.

**Table 3:** Overrepresented motifs identified in promoters of genes involved in fatty acid synthesis, and oleosin and seed storage protein accumulation.

Sequence logo	Matrix ID	TF name	Pathway
	MA1	WRI1	FA synthesis

	MA5	DOF	FA synthesis

	MA40	MYB	FA synthesis

	MA70	GATA	FA synthesis

	MA103	SORLIP5	FA synthesis

	MA117	CBF (LEC1 L1L)	FA synthesis

	MA97	B3-domain (ABI3/VP1)	SSP/Oleosoin

	MA23	bZIP	SSP/Oleosoin

Refer to Additional File 4 for each matrix identifier (ID). The abbreviations of transcription factors (TF), promoter element, and pathway names are as follows: WRI1: WRINLKED 1; DOF: DNA binding with One Finger; SORLIP: Sequences Over-Represented in Light-Induced Promoter; CBF: CCAAT binding factor; bZIP: basic leucine zipper; FA: fatty acid; SSP: seed storage protein.

For the genes and isoforms in the TAG assembly pathway, no overrepresented motifs have been found. Our goal was to identify putative promoter elements that can be used for experimental studies (Additional File 5). Interestingly, promoter motifs for B3 domain TFs, such as ABI3, FUS3 and LEC2, were found to be overrepresented in promoters of genes encoding oleosins and SSPs. Motifs for bZIP factors (e.g., bZIP67) also appeared to be overrepresented in the promoter regions of these genes, but there were no binding matrices for bZIP ABI5 or EEL.

Our approach of computational promoter analysis was limited by the availability of experimentally determined TF-binding sites for deriving binding profiles of additional TFs. We compiled a list of 118 binding matrices for this analysis, but if binding profiles for other TFs can be generated from a reasonable number of known binding sites, we could identify more TFs that possibly regulate the accumulation of seed storage reserves. In addition, we only considered upstream sequences of 1000 bp plus 200 bp 5'-UTR for each gene, because the majority of *cis*-acting regulatory elements are located in this region [74]. Other genomic regions including the 3'-UTR, or even introns, however, can also harbour TF binding sites.

## Conclusions

Our analyses indicate that genes involved in the accumulation of seed storage reserves, along with known *TF *genes, are expressed in distinct patterns during seed maturation. Promoter motifs for CCAAT binding factors LEC1 and L1L, DOF and GATA factors, AP2 WRI1 as well as MYB factors are enriched in the promoter regions of genes involved in FA biosynthesis. Binding sites for B3-domain factors (ABI3/VP1 TF family) and bZIP factors are overrepresented in the promoter regions of genes encoding oleosins and seed storage proteins. When binding profiles for additional TFs become available, more putative regulatory elements will be detected, which in turn can be validated for functionality.

## Methods

### Retrieval and processing of raw hybridization data

The 24 raw hybridization intensity data files (.CEL files) for Arabidopsis seed development were retrieved from The Arabidopsis Information Resource (TAIR) gene expression data repository (http://www.arabidopsis.org/servlets/TairObject?type=hyb_descr_collection&id=1006710873) [71]. Microarray gene expression data analyses were performed using Bioconductor packages [82] in the open-source statistical R environment [83]. The raw data files were imported into Bioconductor using the Simpleaffy package [84]. The hybridization and RNA sample qualities were assessed using a number of quality control metrics (data not shown), and the raw data were background corrected, normalized and transformed to the log_2 _values using the GCRMA package [85]. This normalization method is developed on another normalization approach robust multi-array average (RMA; [86]), and uses probe sequence information (G-C content) for estimating hybridization affinity. The number of genes expressed in seeds was filtered using a log2 value of 6.0 as the cutoff for the binary 'present' or 'absent' calls, and any gene with 'present' calls in less than three samples (corresponding to one seed development stage) was considered as "unexpressed" in these seed samples. After filtering, 12,353 genes expressed in at least one of the eight development stages in developing Arabidopsis seeds were used for subsequent high-level analyses. Custom Perl scripts were written to find the annotation of each gene in the latest CSV file ATH1-121501.na30.annot.csv (November 15 2009) released by Affymetrix for the ATH1 Genome Array and revised in some cases through sequence analysis using BLAST [87]. For example, the TF gene *WRINKLED1 *(AT3G54320) was incorrectly annotated in the Affymetrix file as an aintegumaenta-like protein or ovule development protein aintegumenta (Additional File 1).

### Principal component analysis and association test of global gene expression with seed development

The normalized, log_2_-transformed gene expression data were used for principal component analysis (PCA) using the R prcomp function [83]. For this analysis, expression values of the three replicates for each seed development stage were not combined in order to assess the reproducibility of biological replication. Global testing of the transcriptome with a particular variable (e.g., seed development stage) was carried out using the Globaltest package [51]. This package tests the overall gene expression in group(s) of genes for significant association with a given variable. The test gives one *P*-value for the whole group instead of one *P*-value for each gene to avoid the issue of multiple testing corrections.

### Gene expression correlation analysis and construction of coexpression networks

For the inference of gene coexpression networks in the transcriptome of developing Arabidopsis seeds, we used the 12,353 genes expressed at moderate or high levels and used the Pearson-based correlation coefficient to measure their expression coherence. We first used the median expression data of the genes in the eight samples to compute pairwise correlation coefficients in the R statistical environment, resulting in a correlation matrix of 12353 × 12353. Then we removed self-pairing and duplication, and applied a correlation cutoff of 0.90, which retained over 1.7 million gene pairs representing 11,698 distinct genes for construction of the coexpression network for the Arabidopsis seed genes. This stringent correlation threshold was chosen to eliminate potential spurious correlations in a coexpression network. Network properties were determined using custom scripts. Coexpression networks are visualized using Cytoscape [88]. For time-course clustering analysis, the gene expression values were standardized to have a mean value of zero and a standard deviation of one for each gene profile. This standardization of data ensures that genes with similar temporal profiles are close in Euclidean space during clustering, regardless of their absolute expression levels. The transformed expressions were then clustered using the fuzzy c-means (FCM) clustering algorithm in the Bioconductor Mfuzz package [89]. We determined six clusters can well separate the expression patterns inherent in the dataset, and another FCM parameter m = 1.75, which allows for investigation of the clustering robustness. FCM assigns a membership value in the range of 0-1 for each gene as an indicator of how representative a gene profile is for a specific cluster, and profiles with different membership values were differently coloured.

### Computational analyses of transcription factor binding sites

The genomic sequences 1000 bp upstream plus 200 bp 5' untranslated regions (UTR) for the genes involved in storage reserve biosynthesis were retrieved from the RSAT server [73]. If the intergenic region with the upstream neighbouring gene is <1000 bp long, we only retrieved upstream sequence available in order to prevent using the 3'-end sequence of the adjacent gene in the upstream. Putative TF binding sites on both strands were identified with two software tools, TFBS [76] and fdrMotif [77]. Briefly, the 118 TF binding profiles (position-specific weight matrix, or PWM) were compiled from the literature [27,74] and the JASPAR database [78], and converted into a format suitable for each software tool (Additional File 4). In the TFBS search, an 80% similarity cutoff was adopted. In fdrMotif search, for each input sequence 10 background sequences were generated from a 4th-order Markov model and an upper boundary of false discovery rate (FDR) of 0.15 as suggested by fdrMotif was adopted to control FDR. Only putative binding sites predicted by both tools were retained for subsequent analysis. To ascertain the predictive performance, detected motifs were compared with curated motifs in AtcisDB and AGRIS databases [90,91]. Sequence logos for the predicted motifs for a TF binding profile were created with WebLogo [92].

## Authors' contributions

FYP carried out the data analysis and prepared the first draft of the manuscript. RJW supervised the analysis and revised the manuscript. Both authors read and approved the final version of the manuscript.

## Supplementary Material

Additional File 1 **A list of select genes identified in the subnetwork including the majority of fatty acid biosynthetic genes**. Genes without informative annotation such as hypothetical proteins were excluded. Click here for file

Additional File 2 **A list of select genes identified in the subnetwork including those encoding oleosins and seed storage proteins**. Genes without informative annotation such as hypothetical proteins were excluded. Click here for file

Additional File 3 **The log_2 _expression values of genes involved in storage reserve accumulation across the eight seed development stages**. Genes involved in seed storage reserve accumulation were adopted from early surveys [17,59], and additional genes implicated in this process were identified through sequence analysis using BLAST [87]. Click here for file

Additional File 4 **The 118 high-quality position weight matrices (PWMs) compiled for the analysis**. In each matrix the definition line starts with a '>' sign and an identifier (ID), followed by the description including the transcription factor (TF) name. A row represents a position in the motif sequence, and the four columns represent nucleotides A, C, G, T respectively. Each matrix was standardized such that its frequency sum for the four nucleotides at each position (row) is 1.0000. A blank line was added between every two matrices. One matrix (MA17) was excluded from the file via a manual examination due to ambiguous TF description for the matrix existing in JASPAR [78]. Click here for file

Additional File 5 **Predicted *cis*-acting promoter motifs for genes involved in seed storage reserve accumulation**. The start and end positions of an predicted transcription factor (TF) binding site are relative to the start of each promoter sequence, not to the transcription start site (TSS) of the gene. The numbers +1 and -1 indicate sense and antisense strand, respectively. The score was determined by the transcription factor binding site analysis tool TFBS [76]. Click here for file
